# Robot-assisted, Laparoscopic and Transvaginal Surgery for Pelvic Organ Prolapse: a Comparative Retrospective Single-Center Study

**DOI:** 10.1007/s00192-026-06571-1

**Published:** 2026-03-17

**Authors:** Amerigo Ferrari, Elena Pisacreta, Marta Caretto, Giulia Misasi, Tiziana Fidecicchi, Maria Magdalena Montt-Guevara, Eleonora Russo, Andrea Giannini, Paolo Mannella, Tommaso Simoncini

**Affiliations:** 1 Department of Clinical and Experimental Medicine, Division of Obstetrics and Gynaecology, University of Pisa, Via Roma 67, 56126 Pisa, Italy; 2 MeS (Management and Health) Laboratory, Institute of Management, Scuola Superiore Sant’Anna, Via San Zeno 2, 56127 Pisa, Italy

**Keywords:** Pelvic organ prolapse, Laparoscopy, Robot-assisted surgery, Transvaginal surgery, Operating times, Length of stay

## Abstract

**Introduction and Hypothesis:**

Surgical management for pelvic organ prolapse (POP) is anything but standardized. We aimed to compare operating times, hospital stay, complications, and reinterventions following robot-assisted surgery (RAS), laparoscopy (LPS), or transvaginal surgery (TVS) for POP repair

**Methods:**

We retrospectively analyzed records from our hospital (January 2017–December 2023), including women receiving RAS or LPS (lateral suspension/sacrocolpopexy), or TVS (hysterectomy with POP repair). Cysto/rectopexies without hysterectomy were excluded. Surgical technique was determined from operative descriptions. Outcomes included: 1) operating time; 2) hospital stay; 3) complications; 4) reinterventions. Surgical route was the exposure variable. Adjusted linear, Poisson, logistic, and Cox regressions were used. A sub-analysis on lateral suspension and sacrocolpopexy was performed.

**Results:**

Of 583 women, 14.4% had LPS, 48.5% RAS, and 37.1% TVS. Adjusted models showed longer operating times for LPS vs RAS (+ 21.6 min, 95% CI + 8.7 to + 34.4) and shorter for TVS (-62.7 min, 95% CI -72.9 to -52.5). Hospitalization length was similar for LPS vs RAS, while longer for TVS (+ 0.5 days, 95% CI + 0.3 to + 0.7). Reinterventions and complications did not differ between groups. In sub-analysis, no differences in operating times and length of stay for lateral suspension were observed. For sacrocolpopexy, LPS was longer than RAS (+ 54.1 min, 95% CI + 30.3 to + 77.8); no difference in hospital stay emerged.

**Conclusions:**

RAS and LPS had similar safety and reintervention profiles. LPS was linked to longer operating times than RAS, and similar hospital stay. TVS had shorter operating times but required longer hospitalization. Sub-analyses suggested comparable performance for lateral suspension, while LPS sacrocolpopexy was more time-consuming than RAS.

**Supplementary Information:**

The online version contains supplementary material available at 10.1007/s00192-026-06571-1.

## Introduction

Pelvic organ prolapse (POP) is a condition affecting up to 40% of women, particularly in the postmenopausal population [1, 2]. It often manifests with debilitating pelvic symptoms that compromise daily functioning and well-being. While conservative strategies such as pessaries and physical therapy remain valid for selected patients, surgery is the definitive treatment for most symptomatic or advanced cases, or when explicitly preferred by the patient [3]. Yet, despite the frequency of this condition and the variety of available procedures, surgical management of POP is anything but standardized [4]. The last two decades have witnessed a marked shift toward minimally invasive approaches, with laparoscopy (LPS) and robot-assisted surgery (RAS) gradually replacing traditional open and vaginal procedures in many settings [5]. Abdominal routes, particularly sacrocolpopexy and lateral suspension, have shown favorable outcomes in terms of anatomical correction and durability, especially when mesh reinforcement is employed [6, 7]. However, these techniques vary in complexity, cost, and learning curve. RAS promises enhanced ergonomics and precision, but its real-world benefits over LPS remain debated, particularly given the added resource demands [8, 9]. Meanwhile, transvaginal surgery (TVS) persists as a widely used option due to its low morbidity and reduced operating time, albeit with concerns over higher recurrence and longer recovery in multicompartmental POP [10].

Despite an expanding evidence base, comparative data remain fragmented, often limited to selected procedures or small cohorts. More critically, patterns of surgical care are shaped not only by clinical evidence but by local infrastructure, institutional expertise, and individual surgeon preferences [11]. These differences translated into substantial practice variation [4, 12], both between and within regions, a phenomenon we and others have recently documented in the context of POP surgery.

In this study, we retrospectively compare the three main surgical approaches – robot-assisted, laparoscopic, and transvaginal – for the treatment of pelvic organ prolapse in a high-volume tertiary center. By evaluating operating time, length of stay, perioperative complications, and reintervention rates, we aim to provide a more integrated understanding of the real-world performance of these techniques across a contemporary surgical population.

## Methods

### Study Setting and Design

This was a retrospective study using medical records from an Italian Teaching Hospital. The study period is from January 1, 2017 to December 31, 2023. Our Teaching Hospital is a university tertiary-level maternal-infant care center and a regional referral hub for urogynecological surgery. It handles approximately 1,600–1,700 births annually and houses a multidisciplinary pelvic floor center involving gynecologists, urologists, and proctologists. The hospital also hosts a high-volume robotic surgery center equipped with multiple da Vinci systems, used across various surgical specialties. The hospital complex consists of 21 buildings, with an estimated daily flow of 15,000 people and around 4,800 healthcare professionals, including physicians, nurses, and trainees. As a university institution, our hospital focuses on hospital-based care and academic training and is not integrated with local territorial health services.

### Participants and Outcomes

We included all women with a primary ICD-9-CM (International Classification of Diseases, 9th Revision, Clinical Modification) code for POP who received surgical treatment during the study period. We retrospectively reviewed electronic medical records and, by reading the description of the surgical procedure, we classified the surgical approaches as follows: 1) Robot-assisted surgery (RAS); 2) Laparoscopy (LPS); 3) transvaginal surgery (TVS).

RAS and LPS included abdominal Dubuisson ‘s lateral suspension and sacrocolpopexy: these two techniques were analyzed together in the main analysis to reflect real-world surgical practice and overall performance of minimally invasive abdominal approaches, while a procedure-specific sub-analysis was conducted to differentiate between the two techniques. TVS included vaginal hysterectomy with native tissue pelvic organ prolapse repair according to the McCall culdoplasty technique. Women receiving just cystopexy/rectopexy without hysterectomy were excluded.

Each woman was characterized by sociodemographic and clinical data, such as age, civil status, citizenship, education level, Elixhauser comorbidity index, BMI, concomitant hysterectomy and/or adnexal surgery, post-hysterectomy vault POP, and year. We also revised the operative descriptions of all included surgical procedures to identify three levels of surgical complexity: 1) no intraoperative difficulty; 2) presence of clearly documented adhesions, fibrosis, tissue retraction, or sequelae of previous non-POP surgery deemed to increase technical difficulty; 3) history of previous POP reconstructive surgery. Operative reports were independently reviewed by two coauthors, blinded to surgical outcomes; discrepancies were resolved through discussion to ensure inter-observer agreement.

The primary outcomes were operating times (minutes) and average length of hospital stay (days). Operating times were consistently defined as the interval from skin incision to skin closure, including robotic docking time. Length of hospital stay was computed as the integer number of days between the date of admission and the date of discharge.

The secondary outcomes were complications during hospitalization and reinterventions. We identified complications using ICD-9-CM codes (Supplementary Table S1). We defined reinterventions as repeat surgical procedures performed for POP recurrence or persistence. Follow-up time was calculated from the index surgery date to reintervention or censoring (last available contact or December 31, 2024) and expressed as median (and interquartile range (IQR)). We included women with no missing data for the variables of interest; the proportion of excluded cases due to missing data was small (4.4%).

### Statistical Analysis

The sociodemographic and clinical characteristics of our study population were described as counts and percentages (categorical variables) or mean and standard deviation (continuous variables). We compared these variables between the three surgical groups (RAS, LPS, TVS) by using Pearson's chi-square test for categorical variables and ANOVA for continuous variables.

To explore the effect of the surgical route (exposure variable) on the primary and secondary outcomes, we performed adjusted regression models accounting for the patient differences in sociodemographic and clinical features. Particularly, we used linear regression for operating times, Poisson regression for hospital stays, logistic regression for complications, and Cox regression for reinterventions. Model assumptions were assessed prior to estimation. For linear regression, we checked linearity and the distribution of residuals; for logistic regression, we evaluated model adequacy using standard goodness-of-fit diagnostics; for Poisson regression, we assessed overdispersion using goodness-of-fit statistics; and for Cox regression, we verified the proportional hazards assumption using Schoenfeld residuals.

We also performed two sub-analyses, one on sacrocolpopexy alone, and one on lateral suspension, exploring the impact of the surgical approach on the primary outcomes. Regression models were estimated after verifying that the underlying assumptions were satisfied.

Data management was performed in SAS Software (SAS Institute Inc., Cary, NC, USA) version 9.4, while statistical analyses on Stata Software version 17.0 (Stata-Corp, LLC, College Station, Texas, USA). Statistical significance was set at a p-value less than 0.05.

This study received approval from the Ethics Committee of our Institution.

## Results

We obtained a population of 583 women who were operated on for POP in our Teaching Hospital over a period of seven years. Among them, 84 (14.4%) received LPS, 283 (48.5%) RAS, and 216 (37.1%) TVS. As for RAS cases, 53.4% were lateral suspensions and 46.5% sacrocolpopexies; for LPS, 61.9% were lateral suspensions and 38.1% sacrocolpopexies. The median follow-up time was 1476.0 (740.0—2167.5) days for LPS, 1881.0 (784.0—2399.0) days for RAS, and 2106.0 (1290.0—2541.5) days for TVS.

Mean age was 67.8 (± 9.0) years, with women receiving TVS being significantly older (Table 1). The mean Elixhauser comorbidity index was 0.1 (± 0.3), significantly higher in the TVS group. Mean BMI was 30.8 kg/m^2^, higher in the RAS group. Women living with a partner were 74.8%, with no differences between groups. Non-Italian women were 3.6% of the total, with no between-group differences. Education level did not differ between groups, with 34.5% of women having at least a high-school diploma. Concomitant hysterectomy was performed in 44% of laparoscopic procedures and 41% of robotic procedures. Moreover, 55.1% of women received adnexal surgery, with a predominance in TVS (68%) compared to LPS/RAS (~ 50%). Vault prolapse was present in 11% of laparoscopic cases and 18% of robotic cases. Moderate and high surgical complexity was described in 35% and 4% of interventions, respectively, and more frequently in the RAS group.

**Table 1:** Characteristics of the study population

	LPS	RAS	TVS	TOT	*p*-value
	(*n* = 84)	(*n* = 283)	(*n* = 216)	(*n* = 583)	
Age, mean (SD)	66.9 (8.9)	67.0 (8.9)	69.2 (9.0)	67.8 (9.0)	**0.016**
Elixhauser index, mean (SD)	0.1 (0.3)	0.1 (0.3)	0.2 (0.4)	0.1 (0.3)	** < 0.001**
BMI, mean (SD)	27.2 (2.4)	32.6 (2.4)	29.9 (2.6)	30.8 (3.1)	** < 0.001**
Civil status, n (%)					0.320
With partner	67 (79.8%)	214 (75.6%)	155 (71.8%)	436 (74.8%)	
Without partner	17 (20.2%)	69 (24.4%)	61 (28.2%)	147 (25.2%)	
Nationality, n (%)					0.600
Italian	80 (95.2%)	275 (97.2%)	207 (95.8%)	562 (96.4%)	
Non-Italian	4 (4.8%)	8 (2.8%)	9 (4.2%)	21 (3.6%)	
Education level, n (%)					0.170
Elementary/middle school	48 (57.1%)	193 (68.2%)	141 (65.3%)	382 (65.5%)	
High school/degree	36 (42.9%)	90 (31.8%)	75 (34.7%)	201 (34.5%)	
Years, n (%)					** < 0.001**
2017–2019	29 (34.5%)	160 (56.5%)	131 (60.6%)	320 (54.9%)	
2020–2021	22 (26.2%)	40 (14.1%)	50 (23.1%)	112 (19.2%)	
2022–2023	33 (39.3%)	83 (29.3%)	35 (16.2%)	151 (25.9%)	
Concomitant hysterectomy, n (%)	37 (44.0%)	115 (40.6%)	216 (100.0%)	368 (63.1%)	** < 0.001**
Concomitant adnexal surgery, n (%)	42 (50.0%)	133 (47.0%)	146 (67.6%)	321 (55.1%)	** < 0.001**
Vault prolapse, n (%)	9 (10.7%)	50 (17.7%)	0 (0.0%)	59 (10.1%)	** < 0.001**
Surgical complexity, n (%)					** < 0.001**
Low	55 (65.5%)	148 (52.3%)	154 (71.3%)	357 (61.2%)	
Moderate	26 (31.0%)	119 (42.0%)	61 (28.2%)	206 (35.3%)	
High	3 (3.6%)	16 (5.7%)	1 (0.5%)	20 (3.5%)	
Operating times, mean (SD)	160.1 (52.7)	147.1 (51.9)	95.6 (29.5)	129.9 (52.3)	** < 0.001**
Length of stay, mean (SD)	1.3 (0.6)	1.7 (0.8)	2.3 (1.8)	1.9 (1.3)	** < 0.001**
Reinterventions, n (%)	3 (3.6%)	14 (4.9%)	4 (1.9%)	21 (3.6%)	0.180
Complications, n (%)	7 (8.3%)	10 (3.5%)	5 (2.3%)	22 (3.8%)	**0.047**

*n *number, *%* percentage, *SD* standard deviation, *LPS* laparoscopy, *RAS* robot-assisted surgery, *TVS* transvaginal surgery

Bold values represent statistically significant *p*-values

Mean operating times were 160.1 (LPS), 147.1 (RAS), and 95.6 (TVS) minutes (Table 1). Average hospital stay was 1.3 (LPS), 1.7 (RAS), and 2.3 (TVS) days. Reintervention rates were 3.6%, 4.9%, and 1.9%, respectively. Complication rates were 8.3%, 3.5%, and 2.3%, respectively.

Linear regression model, adjusted for patients’ characteristics, showed longer operating times for LPS vs RAS (+ 21.6 min, 95% CI + 8.7 to + 34.4) and shorter for TVS vs RAS (−62.7 min, 95% CI −72.9 to −52.5) (Table 2). Other covariates significantly associated with longer operating times were concomitant hysterectomy (+ 33.1 min, 95% CI + 21.1 to + 45.2), adnexal surgery (+ 14.0 min, 95% CI + 5.1 to + 23.0), vaginal vault prolapse (+ 23.7 min, 95% CI + 10.3 to + 37.0), and moderate surgical complexity (+ 11.7 min, 95% CI + 4.3 to + 19.2).

**Table 2:** Adjusted regression models for operating times and length of stay (primary outcomes)

	Operating times			Length of stay		
	Coeff	95% CI	*p*-value	Coeff	95% CI	*p*-value
LPS vs RAS	**21.6**	**8.7 to 34.4**	**0.001**	−0.2	−0.5 to 0.0	0.076
TVS vs RAS	**−62.7**	**−72.9 to −52.5**	** < 0.001**	**0.5**	**0.1 to 0.6**	**0.007**
2020–21 vs 2017–19	−3.6	−12.7 to 5.6	0.443	−0.1	−0.2 to 0.1	0.258
2022–23 vs 2017–19	−1.2	−9.6 to 7.1	0.771	**−0.3**	**−0.5 to −0.2**	** < 0.001**
Age	−0.3	−0.7 to 0.1	0.121	0.0	0.0 to 0.0	0.743
Elixhauser index	−9.2	−19.3 to 1.0	0.077	0.1	−0.1 to 0.2	0.656
BMI	1.1	−0.3 to 2.5	0.127	0.0	0.0 to 0.0	0.280
Without vs with partner	**9.2**	**1.2 to 17.2**	**0.025**	0.0	−0.1 to 0.2	0.755
Non-Italian vs Italian	−5.2	−24.1 to 13.7	0.592	0.1	−0.2 to 0.4	0.598
High vs low education	0.5	−6.9 to 7.9	0.903	0.0	−0.1 to 0.1	0.781
Hysterectomy vs not	**33.1**	**21.1 to 45.2**	** < 0.001**	−0.1	−0.2 to 0.1	0.667
Adnexal surgery vs not	**14.0**	**5.1 to 23.0**	**0.002**	0.1	−0.2 to 0.1	0.696
Vault prolapse vs not	**23.7**	**10.3 to 37.0**	**0.001**	0.0	−0.2 to 0.2	0.993
Moderate vs low surg. compl	**11.7**	**4.3 to 19.2**	**0.002**	0.0	−0.2 to 0.1	0.807
High vs low surg. compl	15.0	−4.8 to 34.8	0.136	0.0	−0.4 to 0.3	0.992
Operating times	-			**0.0**	**0.0 to 0.0**	** < 0.001**
Complication vs not	-			**0.6**	**0.4 to 0.9**	** < 0.001**

*Coeff.* Coefficient, *CI* Confidence Interval, *LPS* laparoscopy, *RAS* robot-assisted surgery, *TVS* transvaginal surgery, *BMI* body mass index

Coefficients represent adjusted differences in operating time (minutes) and length of hospital stay (days) relative to the reference category. Positive coefficients indicate longer operating times or hospital stays, whereas negative coefficients indicate shorter durations. Bold values denote statistically significant associations (p < 0.05), which are described in detail in the Results section

Compared to RAS, LPS did not ensure significantly shorter length of hospital stay, while TVS had longer hospital stay (+ 0.5 days, 95% CI + 0.3 to + 0.7) (Table 2). Average length of stay was 0.3 days shorter (95% CI −0.5 to −0.2) in 2022–23 vs 2017–19. Also, operating times were associated with longer hospital stay, although the effect was negligible (+ 0.003 days). Complications during hospitalization were linked to longer hospital stays (+ 0.6 days, 95% CI + 0.4 to + 0.9).

The risk of reinterventions and complications did not differ significantly between groups (Table 3). Surgical complexity was associated with higher chances of complications (OR 4.8, 95% CI 1.7 to 13.2) (Table 3).

**Table 3:** Adjusted regression models for reinterventions and complications (secondary outcomes)

	Reinterventions			Complications		
	HR	95% CI	*p*-value	OR	95% CI	*p*-value
LPS vs RAS	0.6	0.1 to 3.0	0.507	1.8	0.4 to 8.2	0.432
TVS vs RAS	0.2	0.1 to 1.1	0.062	0.7	0.2 to 2.8	0.604
2020–21 vs 2017–19	1.1	0.4 to 3.6	0.835	1.0	0.3 to 3.2	0.948
2022–23 vs 2017–19	0.8	0.2 to 2.6	0.711	0.6	0.2 to 2.0	0.421
Age	1.0	1.0 to 1.1	0.941	1.0	1.0 to 1.1	0.666
Elixhauser index	0.5	0.1 to 3.6	0.494	1.0	1.0 to 1.0	1.000
BMI	1.0	0.8 to 1.2	0.658	0.9	0.7 to 1.1	0.218
Without vs with partner	0.5	0.1 to 1.7	0.263	1.0	0.4 to 3.0	0.949
Non-Italian vs Italian	2.5	0.3 to 21.7	0.400	1.8	0.2 to 17.1	0.605
High vs low education	1.1	0.4 to 2.7	0.867	1.3	0.5 to 3.3	0.648
Hysterectomy vs not	2.2	0.5 to 9.5	0.310	0.9	0.2 to 4.3	0.885
Adnexal surgery vs not	0.3	0.1 to 1.1	0.068	1.6	0.5 to 5.2	0.425
Vault prolapse vs not	0.5	0.1 to 3.1	0.494	1.9	0.4 to 8.0	0.386
Moderate vs low surg. compl	0.9	0.3 to 2.3	0.781	**4.8**	**1.7 to 13.2**	**0.003**
High vs low surg. compl	3.0	0.5 to 16.6	0.209	2.4	0.2 to 24.3	0.446

*HR* Hazard Ratio, *OR* Odds Ratio, *CI* Confidence Interval, *LPS* laparoscopy, *RAS* robot-assisted surgery, *TVS* transvaginal surgery, *BMI* body mass index

Hazard ratios and odds ratios represent adjusted relative associations with reinterventions and complications, respectively, compared with the reference category. Values > 1 indicate higher hazard/odds of the outcome, whereas values < 1 indicate lower hazard/odds. Bold values denote statistically significant associations (p < 0.05), which are described in detail in the Results section

Crude operating times in lateral suspension were 135.7 (± 36.8) for LPS and 135.9 (± 57.1) for RAS. The multivariate sub-analysis showed no significant differences in either operating times or length of hospital stay for LPS vs RAS (Table 4). As for sacrocolpopexy, LPS required longer crude operating times than RAS (199.8 ± 50.7 vs 160.0 ± 41.8 min); this data was confirmed by adjusted analysis (+ 54.1 min, 95% CI + 30.3 to + 77.8). On the other hand, no difference in hospital stay was found (Table 4).

**Table 4:** Sub-analysis: adjusted coefficients for lateral suspension and sacrocolpopexy

		Operating times			Length of stay		
		Coeff	95% CI	*p*-value	Coeff	95% CI	*p*-value
A) *Lateral suspension*							
	LPS vs RAS	12.4	−11.4 to 36.3	0.321	−0.3	−0.7 to 0.1	0.145
B) *Sacrocolpopexy*							
	LPS vs RAS	**54.1**	**30.3 to 77.8**	** < 0.001**	−0.2	−0.6 to 0.3	0.481

*Coeff.* Coefficient, *CI* Confidence Interval, *LPS* laparoscopy, *RAS* robot-assisted surgery

Bold values denote statistically significant associations (*p* < 0.05)

## Discussion

### Main Findings

In this study, we retrospectively compared three different approaches to prolapse surgery in terms of efficiency and clinical outcomes at a single urogynecological surgical referral center in Tuscany over a seven-year period. Women who underwent vaginal surgery were older and presented with a higher burden of comorbidities; in 68% of these cases, concomitant adnexal surgery was performed. Robotic surgery was more frequently performed in women with higher BMI, lower education level, and predominantly Italian nationality. Laparoscopic surgery was employed in women with a similar age and number of comorbidities as those receiving RAS, but with lower BMI and higher education level. Among women undergoing RAS and LPS, 41% and 44%, respectively, had a concomitant hysterectomy; the proportion undergoing adnexal surgery was comparable between the two groups (~ 50%). RAS was more frequently utilized in cases of vault prolapse (18% in RAS vs. 11% in LPS). Overall, the RAS group exhibited greater surgical complexity.

In the main regression analyses, we grouped lateral suspension and sacrocolpopexy into the single category of minimally invasive abdominal surgery. Our aim was to compare surgical routes and platforms rather than individual techniques, thus providing real-world evidence on the overall comparability of RAS vs LPS. Lateral suspension and sacrocolpopexy share a common abdominal, mesh-based strategy for POP repair and are often considered alternative options in clinical practice. Nevertheless, these procedures differ in technical complexity, indications, and risk profiles, which may introduce procedural heterogeneity and limit direct clinical comparability. To address this issue, we conducted technique-specific regression sub-analyses comparing robotic and laparoscopic surgery separately for each procedure.

Compared with RAS, operating times were significantly longer in the LPS group (+ 22 min) and shorter in the TVS group (−63 min). This difference was not observed specifically in cases of lateral suspension, where operating times were comparable between RAS and LPS. However, a clear difference emerged in sacrocolpopexy, for which adjusted models indicated a mean operating time increase of approximately + 54 min in LPS relative to RAS. Other factors independently associated with a significant prolongation of operating time included concomitant hysterectomy (+ 33 min), adnexal surgery (+ 14 min), presence of vault prolapse (+ 24 min), and moderate surgical complexity (+ 12 min).

Length of hospital stay was significantly longer in the TVS group compared to the RAS group (+ 0.5 days). In contrast, no significant differences in length of stay were observed between robotic and laparoscopic surgery, even when sacrocolpopexy and lateral suspension were analyzed separately. A marginal increase in length of stay was associated with prolonged operating time; however, the factor most strongly influencing length of stay was the occurrence of complications during hospitalization (+ 0.6 days).

No statistically significant differences were found among the three groups regarding the risk of complications or reintervention. The sole factor associated with an increased risk of complications during hospitalization was higher surgical complexity.

In previous literature, transvaginal surgery was found to require shorter operating times than abdominal procedures [13], consistently with our findings. As for abdominal surgery, robot-assisted sacrocolpopexy has been described as more time-consuming than laparoscopic sacrocolpopexy, especially before 2020 and in the context of randomized clinical trials (RCTs). For instance, Paraiso et al. reported in a RCT a + 67-min difference in the robotic group, although they partly attributed this difference to robotic docking and surgeon learning curve [14]. In line with our study, they also found no significant differences in complication rates, symptomatic recurrence, or quality of life outcomes between the two approaches. Importantly, we consistently defined operating time as the interval from skin incision to skin closure; differences in reported operating times across studies may partly reflect heterogeneity in how operative duration is measured.

On the contrary, more recent literature aligns with our findings, showing similar or longer operating times for LPS vs RAS. Zhang et al. observed that laparoscopic sacrocolpopexy was associated with significantly longer crude operating times compared to the robotic approach (+ 22 min), with comparable anatomical success rates, and similar mesh exposure and perioperative complication rates [15]. This confirms our finding of a 53-min adjusted operating time advantage for RAS vs LPS in sacrocolpopexy.

Evangelopoulos et al. similarly noted significantly longer operating times for LPS vs RAS, both in univariate analysis (+ 29 min) and multivariate analysis (+ 24 min) [16]. Also, in their study population, robotic surgery was more frequently selected for complex cases, including recurrent prolapse and patients with prior pelvic surgery. Finally, the most recent RCT published in 2024 by Nilsson et al. revealed a + 65-min difference for LPS vs RAS [17].

As for lateral suspension, there is no reliable comparative data for LPS vs RAS. However, single-arm studies suggest a similar duration for the two approaches, consistently with our work. Simoncini et al. reported average operating times for RAS of 117 min [18], while Giannini et al. of 129 min [19]. A recently published RCT reported mean operating times for LPS of 101 min [20]. In our study, mean operating times were 136 min for both RAS and LPS.

Length of hospital stay for vaginal hysterectomy has been described as similar to abdominal approaches [21], differently from our study where we showed slightly shorter hospitalizations after RAS vs TVS. Consistently with previous literature [22–24], we found no difference in length of stay for RAS vs LPS, even when analyzing sacrocolpopexy and lateral suspension separately.

As in our cohort, several observational studies failed to detect significant differences in complication or reintervention rates between transvaginal and abdominal procedures [13]. As for abdominal procedures, RAS and LPS demonstrated equivalent complication and reintervention rates, as confirmed by several RCTs comparing RAS vs LPS for sacrocolpopexy [14, 17, 25, 26]. Moreover, two meta-analyses of robotic versus laparoscopic sacrocolpopexy confirmed similar mesh exposure rates and no differences in anatomic failure, reoperation, or intraoperative and postoperative complications [27, 28]. Finally, although no direct comparative studies exist between LPS and RAS for lateral suspension, single-arm series show similar complication and reintervention rates between the two approaches [18, 29, 30].

### Limitations

The primary limitation of this study lies in its retrospective observational design, which inherently restricts the ability to establish causal relationships. Although we applied comprehensive regression adjustments to account for known confounding variables, the influence of unmeasured or residual confounders cannot be entirely ruled out. Surgical route selection was not random and may have been influenced by unmeasured determinants (e.g., surgeon experience, detailed prolapse staging, or patient preference), potentially affecting both treatment allocation and outcomes. Accordingly, we did not perform propensity score-based analyses because our primary objective was to compare real-world outcomes across surgical approaches rather than to estimate a causal treatment effect. Additionally, propensity score methods were unlikely to be stable in this setting given the limited number of events for some outcomes (particularly reinterventions), the three-group comparison, and the risk of model misspecification when deriving propensity scores from routinely collected administrative data. Multivariable adjustment and procedure-specific sub-analyses addressed the main measured confounders, making additional propensity score methods unlikely to materially change the observed associations.

An additional concern pertains to the quality and consistency of the data, which may be affected by potential inaccuracies or errors in data compilation and reporting. Furthermore, while each Italian region operates with a degree of legislative, fiscal, and political autonomy, caution must be exercised when attempting to generalize these findings to the broader national context, as regional differences may limit the applicability of the results across the entire country.

Despite being based on predefined intraoperative criteria and independently assessed by two reviewers, the classification of surgical complexity may retain an element of subjectivity, owing to variability in the completeness and precision of operative notes; also, the low number of cases in the high surgical complexity category may have precluded the achievement of statistical significance. Reinterventions were identified through institutional records, and the possibility of under-capture for procedures performed at other hospitals cannot be entirely excluded; however, in our clinical experience, patients initially treated at our center (a regional referral hub for pelvic floor surgery) tend to return for subsequent surgical management, making this limitation likely minimal. Complications were identified through ICD-9-CM codes and could not be graded by severity (e.g., Clavien–Dindo). Also, frequencies of category-specific complications were not reported because complications were defined at the patient level using multiple ICD-9-CM diagnostic fields, allowing a single patient to contribute to more than one complication category.

Additionally, our study lacks patient-reported data on women’s experiences and subjective outcomes, and standardized anatomical objective outcomes (e.g., Pelvic Organ Prolapse Quantification system). The final limitation is due to the heterogeneity in how operating times are defined across literature. We consistently measured operating times from skin incision to skin closure. Other studies, however, may report only console or procedural time, excluding setup or closure phases, thus making direct comparisons potentially misleading.

### Implications

This study reinforces the idea that no single surgical approach can be universally optimal for pelvic organ prolapse repair. Instead, each approach offers distinct strengths that should be matched thoughtfully to patient characteristics and procedural demands. Vaginal surgery, often overlooked in favor of minimally invasive techniques, remains a pragmatic and efficient choice, particularly in elderly patients or those with significant comorbidities, where shorter operating times may translate into lower perioperative risk.

Notably, despite shorter operating times, TVS was associated with a longer hospital stay even after adjustment. This finding may reflect differences in postoperative management pathways rather than operative duration alone: in our institution, catheter removal after LPS/RAS is routinely performed immediately after the intervention or on the first postoperative day according to standardized nursing protocols. After TVS, catheter removal is more often guided by individualized clinical indication and may be delayed when early mobilization is limited, and patients are not yet able to ambulate safely to void. In addition, in our institution vaginal packing is commonly used after transvaginal surgery and may further postpone catheter removal thus delaying hospital discharge timing, particularly in older or more vulnerable patients.

In the comparison between abdominal techniques, RAS and LPS appear equivalent in terms of safety, complications, reintervention rates, and overall length of stay. While early data historically associated robotic surgery with prolonged operating times, our findings – consistently with recent evidence – suggest that this gap is narrowing. Especially in anatomically complex procedures like sacrocolpopexy, where dissection near the sacral promontory can be technically challenging, the robotic platform may now offer time-neutral or even time-efficient solutions thanks to its enhanced precision and ergonomics.

Notably, the robotic approach tends to be selected for patients with higher BMI and greater surgical complexity. Despite this, its outcomes remain comparable to those of conventional laparoscopy, thus supporting its validity as an effective alternative in overweight and obese patients who received previous gynecological surgery and/or prolapse repair. While concerns about higher costs persist, there are reasons for cautious optimism: operating times and recovery appear to be improving with experience as surgeons become more proficient, while the growing number of robotic platforms entering the market may introduce long-term downward pressure on costs through competition.

Ultimately, our study supports a personalized model of surgical care. Vaginal, laparoscopic, and robotic techniques each have a valid and complementary role in modern prolapse surgery. The challenge (and opportunity) for clinicians lies in selecting the approach that best aligns with the patient’s anatomical, functional, and personal needs, while also considering the technical nuances of the case. A tailored strategy, rather than a one-size-fits-all approach, is most likely to ensure procedural success and improve patient-centered outcomes.

## Conclusion

In this large retrospective study, we compared transvaginal, laparoscopic, and robot-assisted surgical approaches for pelvic organ prolapse across a contemporary surgical population. Our findings confirm that all three techniques offer comparable safety profiles and reintervention rates, but differ in operating times, hospitalization length, and patterns of use. Vaginal surgery remains a valuable option in older, comorbid patients, offering shorter operating times but longer hospital stays. Robot-assisted and laparoscopic approaches show similar perioperative safety and reintervention profiles; however, robotic surgery is more frequently adopted in patients with higher BMI and greater surgical complexity. Notably, while robotic sacrocolpopexy was once associated with longer operating times, it now appears to offer efficiency advantages in technically demanding procedures. Our findings support a patient-centered, individualized approach to prolapse surgery, where surgical strategy is aligned with anatomical and clinical complexity rather than dictated by a single preferred route.

## Supplementary Information

Below is the link to the electronic supplementary material.

Supplementary file1 (DOCX 105 KB)

## Data Availability

Individual-level data that support the findings of this article cannot be made publicly available because they belong to Azienda Ospedaliera Universitaria Pisana. Aggregated data are available upon reasonable request from the corresponding author.
