# Supplementary material for: Robot-assisted, Laparoscopic and Transvaginal Surgery for Pelvic Organ Prolapse: a Comparative Retrospective Single-Center Study
Source: Int Urogynecol J. 2026 Mar 17;37(8):2497–505. doi: 10.1007/s00192-026-06571-1 (PMC13486119; doi:10.1007/s00192-026-06571-1)
Supplement: Supplementary file 1 [file 192_2026_6571_MOESM1_ESM.docx]

**Robot-assisted, laparoscopic and transvaginal surgery for pelvic organ prolapse: a comparative retrospective single-center study**

**SUPPLEMENTARY MATERIAL**

| **Supplementary Table S1.** ICD-9-CM diagnosis codes used to define complications | | |
| --- | --- | --- |
| **Clinical group** | **ICD-9-CM codes** | **Description** |
| Cardiorespiratory / shock | 5185 | Other pulmonary insufficiency following trauma and surgery |
|  | 9980 | Postoperative shock, unspecified |
| Abdominal complications | 56721 | Peritonitis (acute) generalized |
|  | 56723 | Spontaneous bacterial peritonitis |
|  | 56729 | Other suppurative peritonitis |
|  | 6191 | Digestive-genital tract fistula, female |
|  | 56881 | Hemoperitoneum (nontraumatic) |
| Genitourinary complications | 5933 | Stricture or kinking of ureter |
|  | 5934 | Other ureteric obstruction |
|  | 59382 | Ureteral fistula |
|  | 59653 | Paralysis of bladder |
|  | 5966 | Rupture of bladder, nontraumatic |
|  | 5968 | Other specified disorders of bladder |
|  | 5969 | Unspecified disorder of bladder |
|  | 59960 | Urinary obstruction, unspecified |
|  | 59969 | Urinary obstruction, not elsewhere classified |
|  | 6190 | Urinary-genital tract fistula, female |
|  | 7538 | Other specified anomalies of bladder and urethra |
|  | 78820 | Retention of urine, unspecified |
|  | 78821 | Incomplete bladder emptying |
|  | 78829 | Other specified retention of urine |
|  | 99676 | Other complications due to genitourinary device, implant, and graft |
|  | 5962 | Vesical fistula, not elsewhere classified |
|  | 5982 | Postoperative urethral stricture |
|  | 5988 | Other specified causes of urethral stricture |
|  | 5989 | Urethral stricture, unspecified |
|  | 6192 | Genital tract-skin fistula, female |
|  | 6199 | Unspecified fistula involving female genital tract |
|  | 9986 | Persistent postoperative fistula |
|  | 8670 | Injury to bladder and urethra, without mention of open wound into cavity |
|  | 8671 | Injury to bladder and urethra, with open wound into cavity |
|  | 8672 | Injury to ureter, without mention of open wound into cavity |
|  | 8673 | Injury to ureter, with open wound into cavity |
|  | 6236 | Vaginal hematoma |
| Hemorrhage / anemia / transfusion | 2800 | Iron deficiency anemia secondary to blood loss |
|  | 2809 | Iron deficiency anemia, unspecified |
|  | 2851 | Acute posthemorrhagic anemia |
|  | 5967 | Hemorrhage into bladder wall |
|  | 99811 | Hemorrhage complicating a procedure |
|  | 99812 | Hematoma complicating a procedure |
|  | 99813 | Seroma complicating a procedure |
|  | V582 | Blood transfusion, without reported diagnosis |
| Infection / fever / wound-related | 56722 | Peritoneal abscess |
|  | 7806 | Fever |
|  | 99660 | Infection and inflammatory reaction due to unspecified device, implant, and graft |
|  | 99665 | Infection and inflammatory reaction due to other genitourinary device, implant, and graft |
|  | 99669 | Infection and inflammatory reaction due to other internal prosthetic device, implant, and graft |
|  | 99831 | Disruption of internal operation (surgical) wound |
|  | 99832 | Disruption of external operation (surgical) wound |
|  | 99859 | Other postoperative infection |
|  | 99883 | Non-healing surgical wound |
|  | 99851 | Infected postoperative seroma |
| Other / administrative | 5644 | Other postoperative functional disorders |
|  | 99670 | Other complications due to unspecified device, implant, and graft |
|  | 99881 | Emphysema (subcutaneous) (surgical) resulting from procedure |
|  | 99889 | Other specified complications of procedures not elsewhere classified |
|  | 9989 | Unspecified complication of procedure, not elsewhere classified |
| *Alternate and simplified grouping of complication codes for clinical interpretability*   - Urinary and ureteral injuries: 867x, 593x, 596x, 619x - Cardiorespiratory: 5185, 9980 - Hemorrhagic complications, anemia, and blood transfusion: 99811–99813, 280x, 2851, V582 - Surgical site and device-related infections: 9983x, 9985x, 9966x - Urinary retention and obstruction: 7882x, 5996x - Peritonitis, intra-abdominal abscess, and hemoperitoneum: 5672x, 56881 - Vaginal hematoma: 6236 | | |

| **Supplementary Table S2.** Details on minimally invasive procedures | | | | | |
| --- | --- | --- | --- | --- | --- |
|  | **Total**,  n (%) |  | **Lateral suspension**,  n (%) |  | **Sacrocolpopexy**,  n (%) |
| *Robot-assisted surgery* | 283 (100%) |  | 151 (53%) |  | 132 (47%) |
| Concomitant hysterectomy | 115 (41%) |  | 30 (20%) |  | 85 (64%) |
| Concomitant adnexal surgery | 133 (47%) |  | 41 (27%) |  | 92 (70%) |
|  |  |  |  |  |  |
| *Laparoscopic surgery* | 84 (100%) |  | 52 (62%) |  | 32 (38%) |
| Concomitant hysterectomy | 37 (44%) |  | 10 (19%) |  | 27 (84%) |
| Concomitant adnexal surgery | 42 (50%) |  | 16 (31%) |  | 26 (81%) |
| *Legend*. This table details the distribution of abdominal minimally invasive interventions and concomitant procedures. Values are reported as number (percentage). Percentages for lateral suspension and sacrocolpopexy are calculated over the total number of minimally invasive interventions within each surgical approach (robot-assisted surgery or laparoscopy). For concomitant hysterectomy and adnexal surgery, percentages in the first column are calculated over the total number of interventions within each approach, whereas percentages in the lateral suspension and sacrocolpopexy columns are calculated within each surgical technique. | | | | | |

**Supplementary Figure S1.** Study flowchart

**Supplementary Figure S2.** Kaplan-Meier curve for reinterventions
